# Theory of Mind and physical bullying in preschool children: the role of peer rejection and gender differences

**DOI:** 10.1186/s41155-024-00313-2

**Published:** 2024-07-29

**Authors:** Yanfang Zhou, Xiaojie Deng, Sihui Wang, Leishan Shi

**Affiliations:** 1 School of Teacher Education, Shaoxing University, Shaoxing, China; 2 School of Teacher Education, Ningbo University, Ningbo, China; 3 Faculty of Teacher Education, Lishui University, No. 1 Xueyuan Avenue, Liandu District, Lishui City, Zhejiang Province 323000 China; 4 School of Foreign Languages, Wuhan Qingchuan University, Wuhan, China

**Keywords:** Theory of Mind, Perpetration of physical bullying, Peer rejection, Gender differences, Preschool children

## Abstract

**Background:**

Preschool represents the budding and initial stage of bullying behavior, where perpetration of physical bullying predominates as the primary form of bullying. An in-depth understanding of the factors linked to preschool physical bullying behavior is crucial for enabling early prevention and intervention strategies.

**Objective:**

The purpose of this study was to examine the relationship between Theory of Mind and physical bullying behavior in 4–6 years old children in kindergarten and the mediating role of peer rejection and gender in this relationship.

**Methods:**

Data on perpetration of physical bullying and peer rejection were obtained from 310 preschool children (age range = 52–79 months, M = 66.85, SD = 7.04) by the peer nomination method, and their Theory of Mind was measured by the Theory of Mind Development Scale.

**Results:**

The results showed that Theory of Mind negatively predicted perpetration of physical bullying in preschool children and that Theory of Mind was related to perpetration of physical bullying in preschool children only through peer rejection. Boys were also found to have a stronger association between peer rejection and perpetration of physical bullying in preschool children than girls.

**Conclusion:**

Peer rejection may play a mediating role between Theory of Mind and perpetration of physical bullying in preschool children. In addition, the relationship between peer rejection and perpetration of physical bullying appears to be stronger for boys than for girls. This contributes to our understanding of the relationship between Theory of Mind and perpetration of physical bullying in preschool children and has implications for how bullying prevention and intervention can be tailored to the gender of young children.

## Introduction

School bullying is a form of aggressive behavior that intentionally and repeatedly targets an individual perceived to have less strength or power through physical, verbal, or relational attacks (Salmivalli, 2010). It has a lasting negative impact on the psychosocial development and adjustment of children (Vlachou et al., 2011). Bullying in schools is present among preschool children (Salmivalli & Peets, 2018), will continue to develop (Moreno et al., 2021), and should be prevented early (Monks et al., 2021). However, compared to adolescents, fewer studies have been conducted to analyze bullying in depth in preschoolers (Huitsing & Monks, 2018). According to previous research, physical bullying is the most common form of bullying in preschool children (Zhong et al., 2022). Understanding the individual characteristics and interpersonal context that predict perpetration of physical bullying is key to early prevention and intervention in the occurrence of physical bullying in preschool children. In terms of individual characteristics, Theory of Mind (ToM), a key factor in social cognition (Olson et al., 2011), is a significant predictor of bullying in preschool children (Fink et al., 2020; Vlachou et al., 2011) and has been shown to correlate with physical aggression in preschool children (Wang et al., 2023). In terms of the interpersonal context, peer rejection has been shown as a key indicator of low peer status (Van den Berg & Cillessen, 2015), being it both a predictor of bullying and a result of bullying (Ladd & Troop-Gordon, 2003), and studies have found that the peer rejection process contributes to bullying behavior (Hymel & Swearer, 2015). Therefore, ToM and peer rejection may be important factors in understanding preschool children’s participation in perpetration of physical bullying. In addition, according to existing research, peer rejection and perpetration of physical bullying are closely related to gender (Sentse et al., 2015; Zhong et al., 2022). In this study, we explore how ToM and peer rejection contribute to preschool children’s participation in perpetration of physical bullying and whether there are gender differences in this process.

### ToM and perpetration of physical bullying in preschool children

ToM refers to people’s awareness of their own and others’ mental states (e.g., needs, beliefs, intentions, feelings, etc.), from which they make causal understandings and predictions about corresponding behaviors (Happé et al., 1998). ToM can predict bullying behavior in preschoolers (Monks et al., 2005; Werner et al., 2006), but the relationship between the two has been inconsistently concluded. Several studies have shown that bullies have better ToM skills (Renouf et al., 2010), are able to understand the pain and emotional state of the bullied, and have a strong desire to dominate in order to engage in bullying to gain peer status (Gillespie et al., 2018; Sutton et al., 1999). However, other findings have suggested that bullies have poorer ToM in early childhood (Shakoor et al., 2011), particularly in preschool children, where ToM has been consistently and negatively correlated with aggressive behavior (Lane & Bowman, 2021; Wang et al., 2023).

The social skills deficits perspective posits that individuals’ limitations or deficits in understanding social cues, norms, and expectations may pose challenges in forming and maintaining social connections (Baron-Cohen, 2001). Emphasizing the crucial role of ToM, this perspective underscores that the absence of ToM can result in deficits in children’s ability to comprehend and hypothesize about the mental states of others, potentially hindering their accurate understanding of intentions and motivations. This deficit can lead to misunderstandings and conflicts, subsequently triggering aggressive reactions (Crick & Dodge, 1994; Harvey et al., 2001; Wang et al., 2023). Notably, research has demonstrated a significant negative correlation between ToM and physical aggression (O’Toole et al., 2017). Children with poor ToM skills may misinterpret social cues, fail to correctly perceive the intended message of others, and consistently react aggressively (e.g., hitting) to unintended negative outcomes (Crick & Dodge, 1996; Verhoef et al., 2019). Moreover, they may fail to anticipate the adverse impact of their aggressive behavior on others (Austin et al., 2017).

Building upon existing research, it is evident that ToM plays a crucial role in preschool children’s propensity for perpetration of physical bullying, despite varying research findings. This study aims to contribute further evidence to this discourse. In accordance with the previous literature, on the correlation between poor ToM and perpetration of physical bulling, especially in preschool children, and the social skills deficit perspective, we expect that there will be a negative relationship between ToM and perpetration of physical bullying.

### The mediating role of peer rejection

Peer rejection is a risk factor for perpetration of physical bullying (Perry & Ostrov, 2023) and can predict bullying (Vorlíček & Kollerová, 2023). Some research with school-aged children suggest that bullies are popular with their peers (Van der Ploeg et al., 2019), while research with preschool children suggest that bullies are not popular (Camodeca & Coppola, 2018). Moreover, peer rejection may prevent children from learning and using pro-social peer interaction skills that enhance attraction (Reijntjes et al., 2013). According to group dynamics, children use bullying as a means to pursue greater power and status (Menesini & Salmivalli, 2017). Therefore, the process of peer rejection can contribute to bullying behavior (Vorlíček & Kollerová, 2023). Studies of bullying behavior in preschool children have also reported that peer rejection reinforces bullying behavior and that unpopular children are more likely to bully those who do not like them (Kisfalusi et al., 2022).

ToM are social skills that children need to be competent in peer relationships (Hughes, 2011), and its development affects their peer relationships (Slaughter et al., 2015). In the preschool years, children learn to use ToM in social interactions to build and maintain their social relationships (Fink et al., 2014). Preschool children with low-level ToM may be rejected by their peers because they cannot recognize the harm caused by certain behaviors to their peers and use inappropriate behaviors in peer interaction (Monks et al., 2021; Wang et al., 2023).

According to existing literature, peer rejection has been associated with both perpetration of physical bullying and ToM in preschool children. Some researchers suggest that children’s prior negative experiences play a significant role in the relationship between ToM and aggression (Crick & Dodge, 1994). In a longitudinal study conducted by Fink et al. (2020), the role of ToM and social preferences in bullying behavior was examined. The study revealed that lower levels of ToM early in life predicted decreased peer acceptance, which, in turn, indirectly predicted subsequent bullying behavior through diminished peer acceptance.

In summary, peer rejection represents a negative peer experience characterized by lower peer acceptance, while physical bullying behavior is a subtype of aggression. Given these associations, it is plausible that peer rejection may mediate the relationship between ToM and perpetration of physical bullying in preschool children. Consequently, we contend that peer rejection will mediate the link between ToM and perpetration of physical bullying in preschool children.

### Gender difference

Gender is strongly associated with bullying. In a cross-cultural research, gender differences in bullying behavior were found to be consistent (Smith et al., 2018), with boys more likely to be bullies than girls (Camodeca & Coppola, 2018). Increased hostile behavior was also found to be particularly associated with boys in a longitudinal study (Paz et al., 2020). There are also gender differences in the frequency of bullying behavior (Arseneault, 2017), with boys physically bullying behavior and engaging in aggressive behaviors towards their peers more frequently than girls (Vlachou et al., 2011), and preschool-age boys engage in bullying more frequently and more often than preschool-age girls (Camodeca et al., 2015; Ilola et al., 2016).

There are also gender differences in peer rejection. It has been shown that boys have higher peer rejection scores than girls (Sentse et al., 2015), and girls have higher social preference scores than boys (Farina & Belacchi, 2021). However, no gender differences have been found in the ToM of preschool children (Hughes et al., 2011).

In summary, gender is strongly related with both peer rejection and preschool children’s physical bullying behavior, but whether there are also gender differences in the association between peer rejection and preschool children’s physical bullying behavior needs to be further investigated.

### The present study

**Fig. 1:**
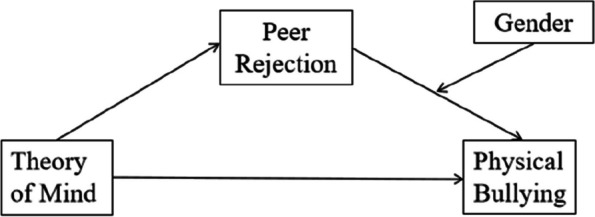
Hypothesized theoretical model

The purpose of this study was to test a model linking ToM to perpetration of physical bullying in preschool children. In contrast to the study by Fink et al. (2020), which focused on bullying behavior in general, this study specifically targeted perpetration of physical bullying, recognized as the most prevalent form of bullying among preschoolers. Additionally, the study aimed to investigate not only the mediating role of peer rejection but also potential gender differences in this mediation process (see Fig. 1), a facet that has not been extensively explored in previous research. Drawing from prior studies, we hypothesized that (1) ToM will be negatively predicted perpetration of physical bullying in preschool children, (2) peer rejection will play a mediating role in the relationship between ToM and perpetration of physical bullying in preschool children, and (3) gender will moderate the association between peer rejection and perpetration of physical bullying in preschool children.

## Method

### Participants

The study included a total of 310 healthy preschoolers who were enrolled in five kindergartens located in Zhejiang Province, China (four in Lishui City and one in Ningbo City). Children in the first year of kindergarten were excluded from the study due to their recent enrollment, limited familiarity with the classroom’s social dynamics, and relatively underdeveloped expressive language skills. The mean age of the participants was 66.85 months (SD = 7.039, range = 52–79 months). Among them, 151 children (49%) were attending their second year of kindergarten, while 157 children (51%) were in their third year. The sample consisted of 159 boys (51.6%) and 149 girls (48.4%).

### Procedure

The study obtained ethical approval from the Ethics Committee of Shaoxing University. Prior to commencing the study, consent was obtained from the kindergarten director, teachers, and their parents. Participants’ teachers were provided with information about the study’s purpose, participated voluntarily, and had the right to withdraw at any time without facing penalties. Participant information and data is kept confidential. The researcher conducted one-on-one tests during classroom breaks. Only the researcher and participants were present during test administration. At the end of each session, participants were escorted back to their head teacher by the researcher.

Measurements were conducted in two waves. First, 911 preschoolers from 29 classrooms were interviewed one-on-one using the peer nomination method to collect information about participants’ physical bullying behavior and peer rejection. To minimize measurement error, all children from each classroom were measured. Each interview in this first wave took approximately 5–8 min per participant. Next, a subset of participants among the 911 children was again selected for the ToM measure. The selection was done by randomly selecting 10–12 children per class from the 911 preschoolers. Not all 911 children were measured in the second wave because this phase of the test was more time-consuming, taking approximately 10–15 min per participant. Ultimately, a total of 310 children completed the physical bullying behavior, peer rejection, and ToM measures. The order in which children participated in the two measurements was randomized and the time interval was approximately 1 week. No one refused to participate or withdraw.

### Measures

#### Sociodemographic information

Basic information about the child, including gender (1 = boy, 2 = girl) and age in months, is provided by the head teacher.

#### ToM

Five tasks from the ToM Development Scale developed by Wellman et al. (2006) were selected for this study: Diverse-desires, Knowledge-ignorance, Contents False-Belief, Explicit false-belief, and Hidden-emotion. The test stories were translated and adapted for Chinese children (Ge et al., 2021). All four tasks scored between 0 and 2, except for the Diverse-desires task, which scored between 0 and 1. ToM’s total score was between 0 and 9.

#### Peer rejection

This study used the peer nomination method to measure peer rejection (Coie et al., 1982). The researcher asked the children one-on-one the question, “Which three children in your class do you dislike to play with the least ?”. Each child can only nominate peers belonging to their classroom; cross-gender nominations are allowed. Finally, as a class, the number of times each child was nominated was converted to a standardized score (Ronchi et al., 2019).

#### Physical bullying in preschool children

This study utilized interview questions from the physical bullying dimension of the Role Assessment developed by Monks et al. (2002). Only the implementation of the perpetration of bullying behavior is assessed, such as, “Which children in the classroom regularly hit/kick/bite people on purpose ?”. The questionnaire has been shown to have good psychometric properties (Lee et al., 2015; Monks & Smith, 2010; Monks et al., 2003). In this study, the Cronbach’s alpha coefficient is 0.896. The questionnaire uses the peer nomination method of one-to-one interviews with preschool children and has been shown to be applicable in groups of preschool children (Camodeca & Coppola, 2018; Camodeca et al., 2015). During the interviews, we consider whether preschool children mention salient features of bullying behavior such as power imbalance, repetition, and intentionality (Moreno et al., 2021). In peer nominations, we recorded the nomination as physical bullying behavior only if the distinctive features of the bullying behavior described above were mentioned, in order to be able to make a distinction between physical bullying and general physical aggression. Finally, taking into account the varying size of each class, we standardized the number of nominations recorded for each child on a class-by-class basis (Camodeca et al., 2015).

### Statistical analysis

First, we performed a preliminary descriptive analysis and bivariate correlation analysis of the data using SPSS 27.0. The study variables included gender, ToM, peer rejection, and physical bullying behavior as shown in Table 1. Second, we constructed the mediating structural equations using Mplus 8.3 and used maximum likelihood (ML) (Satorra & Bentler, 2001) and bootstrap methods for analysis. Structural equation modeling used ToM as the independent variable, peer rejection as the mediating variable, and physical bullying behavior in preschool children as the outcome variable. Finally, we again used Mplus 8.3 to build a mediator with conditioning. Based on the mediating structural equation modeling, gender was added as a moderating variable to test whether there are gender differences between two different groups, boys and girls, on the pathway of peer rejection and perpetration of physical bullying in preschool children.

**Table 1:** Descriptive statistics and correlations among the study variables

	Gender	ToM	Peer rejection	Physical bullying
Gender	1			
ToM	.062	1		
Peer rejection	− .287^***^	− .171^**^	1	
Physical bullying	− .224^***^	− .157^**^	.704^***^	1
M		7.95	.035	.016
SD		1.45	.959	1.06

^*^p < 0.05,^**^p < 0.01, ^***^p < 0.001

We used several metrics to analyze the fit of the model. If confirmatory fit index (CFI) and Tucker-Lewis index (TLI) are greater than 0.90, the data are considered well fitted (Hu & Bentler, 1999). Root Mean Square Error of Approximation (RMSEA) < 0.08 indicates that the model is fitted precisely (Browne & Cudeck, 1993). Standardized root mean square residual (SRMR) < 0.08, then the model is considered well fitted (Hu & Bentler, 1999).

## Results

### Descriptive statistics and correlations

As shown in Table 1, the results of the correlation analysis showed that ToM was significantly and negatively related with both peer rejection (*r* =  − 0.171, *p* < 0.01) and perpetration of physical bullying in preschool children (*r* =  − 0.157, *p* < 0.01). Peer rejection was significantly and positively related with perpetration of physical bullying in preschool children(*r* = 0.704, *p* < 0.001). Gender was significantly and negatively correlated with peer rejection (*r* =  − 0.287, *p* < 0.001) and perpetration of physical bullying in preschool children (*r* =  − 0.224, *p* < 0.001), but not with ToM. With this, male preschoolers demonstrate high levels of peer rejection and physical bullying behavior.

### Mediation effect test

Due to the significant correlations between ToM, peer rejection, and physical bullying behavior in preschool children, further tests of mediation effect can be conducted. A structural equation model (M_1_) was constructed using Mplus 8.3 with physical bullying behavior in preschool children as the outcome variable, ToM as the independent variable, and peer rejection as the mediating variable. The model fits well, CFI/TLI = 1.000, RMSEA = 0.000, SRMR = 0.000.

The results found that ToM negatively predicted perpetration of physical bullying in preschool children (*β* =  − 0.157, *p* < 0.01), and the total model effect was significant, supporting hypothesis 1. Mediation effect results indicated (see M_1_ in Table 2) that ToM negatively predicted peer rejection (*β* =  − 0.171, *p* < 0.01) and peer rejection positively predicted perpetration of physical bullying in preschool children (*β* = 0.675, *p* < 0.001). The mediation effect was − 0.115 (*p* < 0.01), with a 95% confidence interval of [− 0.213, − 0.018], not including 0. Conditional indirect effects can be deemed to be significant when 95% confidence limit (CI) does not include 0 (Hayes, 2013). The direct effect of ToM and preschool children’s physical bullying behavior was no longer significant (*β* =  − 0.042, *p* = 0.320), indicating that peer rejection fully mediated the association between ToM and preschool children’s physical bullying behavior, supporting the hypothesis 2.

**Table 2:** Direct and indirect effects between ToM and physical bullying in preschool children

	M_1_			M_2_			M_3_		
	Direct effect		Indirect effect	Direct effect		Indirect effect	Direct effect		Indirect effect
	Peer rejection	Physical bullying	Estimate	Peer rejection	Physical bullying	Estimate	Peer rejection	Physical bullying	Estimate
ToM	− 0.171 (− 0.109)^**^	− 0.042 (− 0.029)		− 0.196 (− 0.136)^*^	− 0.039 (− 0.030)		− 0.089 (− 0.042)	− 0.045 (− 0.024)	
Peer rejection		0.675 (0.734)^***^			0.707 (0.781)^***^			0.490 (0.545)^***^	
ToM→ Peer rejection→ Physical bullying			− 0.115 (− 0.080)^**^			− 0.138 (− 0.106)^*^			− 0.044 (− 0.023)

Unstandardized regression coefficients or mediated effect sizes are in parentheses

^*^p < 0.05,^**^p < 0.01,^***^p < 0.001

### Gender differences

In accordance with hypothesis 3, we further included gender in the mediated structural equation model to explore whether boys (M_2_) and girls (M_3_) differed in the pathway between peer rejection and perpetration of physical bullying in preschool children (see Fig. 1). Thus, we built a mediated structural equation model with moderation by adding gender as a moderator variable. The model fits well, CFI/TLI = 1.000, RMSEA = 0.000, SRMR = 0.000.

After testing (see M_2_ and M_3_ in Table 2), there was a significant difference between males and females on the pathway between peer rejection and perpetration of physical bullying in preschool children (*b*_males_ = 0.781, *p* < 0.001, *b*_females_ = 0.545, *p* < 0.001, *b*_males_ − *b*_females_ = 0.236, *p* < 0.05), and males were higher than females. This suggested that the association between peer rejection and perpetration of physical bullying in preschool children is stronger for boys than for girls. Research hypothesis 3 was supported.

## Discussion

Our study tested the proposed theoretical model and the results supported the assumptions of the theoretical model. ToM negatively predicted perpetration of physical bullying in preschool children, that is, a lower level of ToM indicated a higher level of perpetration of physical bullying in preschool children. Moreover, ToM was associated with perpetration of physical bullying in preschool children exclusively through peer rejection. In addition, we found that in preschool children, boys had stronger associations with peer rejection and perpetration of physical bullying than girls.

### ToM and perpetration of physical bullying in preschool children

This study found that ToM negatively predicted perpetration of physical bullying in preschool children, which is consistent with existing research findings (Lane & Bowman, 2021; Shakoor et al., 2011; Wang et al., 2023). This could provide further evidence for the theoretical explanations of the social skills deficit perspective proposed by Crick and Dodge (1994). The lower a preschool child’s level of ToM, the less cognitively aware he or she is of social information, which leads to preschool children often failing to meet their own needs or correctly perceive each other’s internal mental states in peer interactions. After misinterpreting social information, they may use physical bullying behavior as a response, and perpetration of physical bullying is the most common form of bullying among preschool children (Verhoef et al., 2019).

Although a positive relationship between ToM and perpetration of physical bullying has also been found in previous studies, these include only older children. Even after controlling for age and language ability, 7-to-10-year-old bullies have higher levels of ToM than non-bullies of the same age (Sutton et al., 1999). It may be that they are dealing with more complex social situations (Austin et al., 2017), need to better understand the psychological states of others to support social relational behaviors (Imuta et al., 2016), or even use higher levels of ToM to effectively engage with indirect forms of bullying, such as relational bullying (Gillespie et al., 2018). This suggests that the nature of the relationship between ToM and perpetration of physical bullying may be age-related and that bullies in preschool age may generally have lower ToM.

### The mediating role of peer rejection

To uncover the process by which ToM influences perpetration of physical bullying in preschool children, we tested the mediating role of peer rejection. The results found that ToM of preschool children was a significant predictor of peer rejection, which is consistent with the results of existing research (Badenes et al., 2000). The development of ToM in preschool children affects peer relationships (Slaughter et al., 2015). Preschool children with low levels of ToM are unable to perceive the needs, beliefs, and emotional states of the other people in peer interactions in a timely manner and are not aware of how some of their behaviors may harm the other people, thus exacerbating peer rejection.

In addition, this study found that peer rejection was a significant predictor of physical bullying behavior in preschool children and was highly positively related with bullying behavior. This is consistent with existing research findings (Vorlíček & Kollerová, 2023). Peer rejection prevent preschool children from acquiring pro-social interaction skills in peer interactions, and they tend to use inappropriate means to solve problems or satisfy their own desires and needs when they are in conflict with their peers, thus increasing the likelihood of perpetration of physical bullying.

In conclusion, similar to existing findings, low levels of ToM can indirectly predict bullying through poorer peer acceptance (Fink et al., 2020). However, the present study focuses on physical bullying, the most common type of bullying behavior among preschoolers, and supports the idea that ToM is related to preschool children’s physical bullying behavior exclusively through the role of peer rejection.

### Gender differences

It was found that the boys’ group had a stronger relationship between peer rejection and perpetration of physical bullying in preschool children than the girls’ group, which is consistent with the findings of existing studies (Farina & Belacchi, 2021; Monks et al., 2021; Zhong et al., 2022). This may be due to the fact that girls are less likely than boys to engage in melee, competitive activities and physical aggression (Smith et al., 2018). In peer interactions, girls tend to solve problems in a cooperative, communicative manner (Rose & Smith, 2018) and thus less likely to be rejected by their peers, while boys are more likely to use physical violence to resolve conflict (Ilola et al., 2016). Boys have a greater appreciation for strength and status in the peer group and are expected to appear strong, imposing, and powerful. Therefore, when they face rejection from their peers, they may demonstrate their dominance by exerting physical bullying behavior to gain respect and submission. And they are more interested in the approval of their classmates of the same sex and less concerned about rejection by others (Kisfalusi et al., 2022). However, this does not mean that girls do not engage in perpetration of physical bullying or do not display challenging behavior in the face of peer rejection. It is just that overall, when faced with peer rejection, boys are much more likely than girls to resort to perpetration of physical bullying to resolve conflict.

### Limitations and future directions

Several limitations of the current study should be considered when interpreting the results and guiding future research. Firstly, due to time constraints, we only did a cross-sectional study to explore the factors associated with perpetration of physical bullying from the preschoolers’ ToM only, without focusing on the factors associated with family, teacher, and classroom environments, as well as controlling for other relevant confounding variables. Future research could attempt to develop a multilevel model or longitudinal design to comprehensively explore the factors influencing preschool children’s physical bullying behavior and to consider controlling for preschoolers’ cognitive deficits, externalizing problems, and other relevant confounding variables. Second, the sample of this study was drawn only from urban kindergartens in Zhejiang Province, China, which lacked diversity. There are differences in the level of economic development, education level, and educational environment in different regions, and further investigation is needed to determine whether the findings of this study are stable. Future research could focus on provinces or regions with different levels of development and survey urban and rural kindergartens to analyze whether there are differences in bullying. Third, the data collected for this study were obtained from preschool children, using the peer nomination method to obtain perpetration of physical bullying results. Although studies have demonstrated that British children between the ages of 4 and 6 are able to reliably nominate their peers in the roles of bully, victim and protector (Monks et al., 2003), assessing bullying behavior in preschoolers is often a difficult and complex process. Future research could attempt to incorporate the perspectives of different groups and use multiple measures that incorporate children’s developmental characteristics and unique age-related behavioral patterns to construct better measurement techniques for assessing bullying behavior in preschool children (Vlachou et al., 2011). Finally, future research may more comprehensively examine the forms of child bullying (such as physical, verbal, and relational bullying) and explore whether there are differences between different age groups, especially when considering gender differences.

### Implications for theory and applications

The results of this study contribute to our understanding of the relationship between ToM and perpetration of physical bullying in preschool children, as well as the mediating role of peer rejection and gender differences, enriching related research. In addition to these theoretical implications, our findings have important practical implications for early intervention in physical bullying in preschool children. First, helping preschool children to improve their level of ToM so that they can understand the feelings of others in their interactions enhance their social skills, which might be an effective way to reduce perpetration of physical bullying in preschool children. For example, therapists might directly target these social skills and provide some models of therapy such as mindfulness or mentalization-based treatments. At home, parents can also help children develop an adequate understanding of their own and others mental states, through positive parenting. Second, preschool children are nurtured to be inclusive and fair, minimizing exclusion and rejection of peers (Vorlíček & Kollerová, 2023). Forming positive, equal peer relationships and creating a positive, respectful, and supportive classroom climate reduces the likelihood of perpetration of physical bullying in preschool children (Balvin & Christie, 2020). Finally, educators need to take targeted measures to guide boys and girls to interpret social cues in a more appropriate and pro-social way (for example, by reducing hostile bias in ambiguous situations) and help them gradually construct meaning in social interactions. Especially for boys, educators can use role-playing and other methods to teach them to learn effective communication skills in peer interactions and enhance peer acceptance, while reducing the occurrence of physical bullying behavior. In addition, educators should emphasize that both boys and girls have a responsibility not to participate in any form of bullying, stimulate intolerant attitudes towards bullying behavior, and encourage cooperation and mutual assistance among peers.

## Conclusion

In summary, our findings suggest that ToM negatively predicts perpetration of physical bullying in preschool children and that ToM was related to perpetration of physical bullying in preschool children only through peer rejection as well as that boys are stronger than girls in the relationship between peer rejection and perpetration of physical bullying in preschool children. These results have important implications for early prevention and intervention of bullying in preschool children, for emphasizing the development of ToM in preschool children, and for improving preschool children’s peer relationships, especially boys.

## Data Availability

The data that support the findings of this study are available from the corresponding author, Leishan Shi, upon reasonable request.

## Abbreviations

ToM: Theory of Mind
ML: Maximum likelihood
CFI: Confirmatory fit index
TLI: Tucker-Lewis index
RMSEA: Root Mean Square Error of Approximation
SRMR: Standardized root mean square residual
CI: Confidence limit
